# Eye-movement-based machine learning for identifying Parkinson’s disease dementia in a retrospective cohort: an interpretable model-development study

**DOI:** 10.3389/fnagi.2026.1935091

**Published:** 2026-10-01

**Authors:** Jing Wu, Qing Liu, Song Yang, Yuqing Zhang, Yuanlu Shu, Junwen Wang, Min Wu, Tian Tian

**Affiliations:** 1 Department of Neurology, The Affiliated Hospital of Guizhou Medical University, Guiyang, China; 2 Department of Neurology, The Second People’s Hospital of Guiyang (Jinyang Hospital), The Affiliated Jinyang Hospital of Guizhou Medical University, Guiyang, China; 3 Department of Neurology, The Affiliated Xingyi Hospital of Guizhou Medical University (Xingyi People’s Hospital), Xingyi, China; 4 Department of Psychosomatic Diseases, The Second People’s Hospital of Guizhou, Guiyang, China; 5 Department of Neurosurgery, Guizhou Provincial People’s Hospital, Guiyang, China; 6 Key Laboratory of Human Brain Bank for Functions and Diseases, Department of Education of Guizhou Province, Guiyang, China

**Keywords:** cognitive screening, eye movement, machine learning, Parkinson’s disease dementia (PDD), support vector machine (SVM)

## Abstract

**Background:**

Parkinson’s disease dementia (PDD) is a major non-motor complication of Parkinson’s disease (PD), but conventional cognitive screening is influenced by education, language, motor impairment, fatigue, and testing conditions. We developed and interpreted machine-learning models using eye-movement and demographic features to identify PDD among patients with PD.

**Methods:**

In this retrospective single-center study, 486 patients were screened between December 2024 and February 2025. After exclusions, 297 patients with idiopathic PD were analyzed, including 84 without dementia and 213 with PDD. Thirteen machine-learning algorithms were trained using demographic and task-derived eye-movement variables. Performance was assessed using ROC AUC, PR AUC, classification metrics, calibration, the Brier score, and decision-curve analysis. SHapley Additive exPlanations (SHAP) were used to interpret the final model.

**Results:**

Patients with PDD showed poorer cognitive performance and abnormalities in antisaccade inhibition, saccade execution, and fixation stability. Seven predictors were retained: age, sex, education level, antisaccade accuracy, mean overlap-saccade completion duration, mean gap-saccade velocity, and lateral-gaze deviation duration >4°. Logistic regression and elastic net achieved the highest test-set ROC AUCs (both 0.962), while logistic regression had the lowest Brier score (0.077). A radial basis function support vector machine (SVM) was retained as the primary classifier for detailed evaluation because it provided balanced performance across threshold-dependent metrics and could capture potentially nonlinear relationships and interactions. In the test set, the SVM achieved a ROC AUC of 0.947 and PR AUC of 0.979. Accuracy, sensitivity, specificity, and F1 score were 0.867, 0.870, 0.857, and 0.904, respectively. SHAP identified education, antisaccade accuracy, mean overlap-saccade completion duration, mean gap-saccade velocity, and lateral-gaze deviation duration >4° as the leading contributors.

**Conclusion:**

A seven-feature model combining demographic and eye-movement measures showed promising internal discrimination of PDD from PD without dementia. Eye-movement-based machine learning may complement clinical cognitive assessment, but external validation in larger and more representative cohorts is required before clinical use.

## 1 Introduction

PD is a multisystem disorder, and cognitive decline is an important factor affecting patients’ functional independence and prognosis (Kwon et al., 2014; He et al., 2021). Guidelines for the diagnosis and treatment of PD also recognize PDD as a common non-motor manifestation whose occurrence increases with disease duration, and emphasize that early diagnosis and timely intervention can help preserve social functioning and quality of life (Irwin et al., 2013). Cognitive decline in PD spans a heterogeneous trajectory. It may progress from subtle deficits through PD-related mild cognitive impairment (PD-MCI) to dementia, rather than emerging only in late disease. Accurate identification of PDD among patients with PD is therefore clinically relevant for risk stratification, individualized management, and follow-up planning. Although the MoCA is routinely used in clinical practice, its scores can reflect educational and linguistic background, motor limitations, fatigue, and the circumstances of testing (Dalrymple-Alford et al., 2010; Coen et al., 2016; Elkana et al., 2020; Patrick et al., 2024). Behavioral markers that rely less on verbal or motor responses may therefore provide useful information in addition to conventional assessment.

At present, cognitive impairment in PD is mainly evaluated through clinical judgment, brief cognitive screening instruments, and comprehensive neuropsychological assessment (Dalrymple-Alford et al., 2010). Clinical and epidemiological practice commonly uses the Montreal Cognitive Assessment (MoCA) and Mini-Mental State Examination (MMSE). However, brief screens may have ceiling effects, variable sensitivity, and limited resolution of domain-specific cognitive profiles (Jia et al., 2021). Comprehensive neuropsychological assessment provides more detailed cognitive information, but it is time-consuming and difficult to implement repeatedly in routine clinical practice. Other diagnostic approaches, including neuroimaging, cerebrospinal fluid biomarkers, and related biological examinations, can provide useful diagnostic information, although their broader clinical application is constrained by cost, invasiveness, accessibility, and scalability (Abraham et al., 2026; Ay et al., 2026; Erickson et al., 2026). Taken together, these limitations leave a practical need for objective, non-invasive, and clinically feasible tools that can complement conventional cognitive assessment in patients with PD.

Researchers are increasingly evaluating oculomotor measures as digital indicators of cognitive dysfunction across neurodegenerative disorders (Leng et al., 2024). Eye movements recruit distributed cortical and subcortical systems, notably prefrontal, basal-ganglia and frontostriatal circuitry. Dysfunction within these systems also contributes to executive deficits in PD (Zgaljardic et al., 2003; Chan et al., 2005). Across multiple oculomotor paradigms, altered saccades, pursuit and fixation can index deficits in attention, response inhibition, working memory and visuospatial processing (Chan et al., 2005; Riek et al., 2023). In PD populations, eye-movement parameters have been associated with cognitive performance and disease severity, suggesting that eye tracking can provide an objective behavioral readout of neurodegenerative and cognitive changes (Koch et al., 2024). Nevertheless, current evidence indicates that eye-movement measures alone are unlikely to provide sufficient sensitivity or specificity as standalone diagnostic tools; their clinical value may be greater when combined with relevant clinical indicators and advanced analytic methods (Antoniades and Spering, 2024).

Recent eye-tracking research in PD has largely centered on differentiating patients with PD from healthy individuals and describing disease-related oculomotor abnormalities (Antoniades and Spering, 2024; Koch et al., 2024). In contrast, relatively few studies have directly evaluated whether eye-movement features can discriminate cognitively intact PD from PD-MCI or PDD (Brien et al., 2023; Riek et al., 2023).

Machine learning can combine heterogeneous oculomotor measurements and estimate their joint value for individual-level prediction (Hao et al., 2024; Lin et al., 2024). Video-based eye tracking combined with machine learning has been used to classify PD and characterize stages of cognitive dysfunction, suggesting that brief, non-invasive eye-movement tasks could supplement conventional clinical screening (Koch et al., 2024). In broader cognitive impairment research, models incorporating gait and eye-tracking features have shown feasibility for detecting cognitive impairment and subjective cognitive decline (Hao et al., 2024). Studies in PD have also suggested that combining eye-movement and gait features may improve the recognition of PD-related phenotypes compared with single-modality approaches (Li et al., 2024b). Important questions remain regarding which eye-movement features are most informative for identifying PDD among patients with PD, how different machine-learning algorithms perform in this specific classification task, and whether model predictions can be interpreted in a clinically transparent manner. In clinical prediction, SHAP summarizes global feature importance and quantifies the contribution of each variable to individual predictions (Lundberg and Lee, 2017).

The present retrospective cohort study was conducted among patients with PD and PDD at The Second People’s Hospital of Guiyang between December 2024 and February 2025. The predictive features included demographic variables and task-derived eye-movement parameters, including age, sex, education level, antisaccade accuracy, mean overlap-saccade completion duration, mean gap-saccade velocity, andlateral-gaze deviation duration >4°. Several machine-learning algorithms were developed and compared, including K-nearest neighbors, random forest, support vector machine, XGBoost, multilayer perceptron, and stacking models, with SHAP applied for interpretability analysis. This study therefore aimed to develop, compare, and interpret eye-movement-based machine-learning models for identifying PDD among PD.

## 2 Materials and methods

### 2.1 Study design, ethics, and participants

This retrospective cohort study included patients with parkinsonism treated at the Second People’s Hospital of Guiyang between December 2024 and February 2025. PD was diagnosed using the Movement Disorder Society Clinical Diagnostic Criteria for Parkinson’s Disease (Postuma et al., 2015), PDD status was determined based on criteria adapted from the MDS Task Force recommendations for dementia associated with PD (Emre et al., 2007).

Eligibility for the PD-without-dementia group required parkinsonism, defined by bradykinesia plus rest tremor, rigidity, or both. Patients also needed clinically established or probable PD under MDS criteria, absence of dementia at enrollment, and capacity to complete eye-movement testing.

Eligibility for the PDD group required PD to have been diagnosed before the onset of dementia, with dementia developing in the context of PD through an insidious onset and gradual progression. Cognitive impairment was required to involve at least two of four core domains: attention, executive function, visuospatial function, and memory. The cognitive deficits had to interfere with daily functioning independently of motor or autonomic symptoms, and patients were required to be able to complete the eye-movement examination (Emre et al., 2007).

Patients were excluded for atypical or secondary parkinsonism, including multiple system atrophy, progressive supranuclear palsy, corticobasal syndrome, drug-induced parkinsonism, and vascular parkinsonism, or when another disorder provided a better explanation for the clinical presentation. Other neurological exclusions included stroke, epilepsy, brain tumor, traumatic brain injury, normal-pressure hydrocephalus, frontotemporal dementia, and dementia with Lewy bodies when dementia preceded or developed within 1 year of parkinsonism onset. Major psychiatric disorders, delirium, acute confusional states, or severe depression sufficient to account for cognitive impairment were also exclusionary. Additional exclusion criteria comprised severe cardiopulmonary, hepatic, renal, metabolic, or infectious disease likely to affect cognition or eye-movement performance; current alcohol or substance abuse; carbon monoxide poisoning; exposure to drugs associated with parkinsonism or marked ocular motor abnormalities; and inability to provide reliable clinical information.

Additional exclusions from the eye-movement analysis included ocular or neurological conditions that could impair visual acuity, visual fields, or ocular motility, such as optic nerve disease, oculomotor nerve palsy, macular disease, retinal detachment, glaucoma, severe cataract, color blindness, ptosis, strabismus, and pathological nystagmus. Patients were also excluded when severe tremor, dyskinesia, dystonia, postural instability, or excessive head movement prevented stable eye tracking. Other reasons for exclusion were inability to understand or follow task instructions, failed calibration or validation, excessive blinking or missing data, and inadequate eye-tracking data quality.

The Ethics Committee of Guiyang Second People’s Hospital approved the study (No. JYYY-2024-XM-36). All participants, or their legally authorized representatives when appropriate, provided written informed consent. A total of 486 patients were initially screened from the clinical database. After exclusion of patients with non-idiopathic parkinsonism, 325 patients with idiopathic PD remained. Of these, 28 were excluded because of substantial missing data in the variables required for model development. The final analytic cohort therefore comprised 297 patients, all of whom had complete data for the variables included in the modeling workflow and had undergone eye-movement examination.

### 2.2 Demographic and clinical characteristics

Demographic characteristics, medical history, laboratory findings, and clinical assessment data were extracted from the hospital electronic medical record system. Neuropsychological and clinical assessments were performed by trained neurologists as part of routine clinical evaluation. Global cognition was evaluated using the MMSE and MoCA, and visuospatial and executive function using the Clock Drawing Test. The Hamilton Anxiety Rating Scale (HAMA) and Hamilton Depression Rating Scale (HAMD) were used to assess anxiety and depressive symptoms, respectively. Sleep quality was measured using the Pittsburgh Sleep Quality Index (PSQI). PD severity was evaluated using the total score of Parts I–IV of the Movement Disorder Society–sponsored revision of the Unified Parkinson’s Disease Rating Scale (MDS-UPDRS) and the modified Hoehn and Yahr stage. Motor assessments were performed in the OFF medication state.

### 2.3 Magnetic resonance imaging (MRI) acquisition and imaging assessment

All participants underwent brain MRI. The imaging protocol included axial T1-weighted imaging, axial T2-weighted imaging, axial fluid-attenuated inversion recovery (FLAIR) imaging, and coronal T1-weighted imaging. On T2-weighted and FLAIR sequences, white matter hyperintensities were defined by increased signal within cerebral white matter. Periventricular WMHs and deep WMHs were assessed separately using the Fazekas visual rating scale (Fazekas et al., 1987). Medial temporal atrophy (MTA) was visually evaluated on coronal T1-weighted images using the Scheltens MTA scale (Scheltens et al., 1992). Each hemisphere was rated separately on a five-point scale ranging from 0 to 4, with higher scores indicating more severe medial temporal atrophy. For statistical analysis, the higher score of the two hemispheres was used. Global cortical atrophy (GCA) was assessed using the Pasquier visual rating scale (Pasquier et al., 1996). Two trained neurologists independently rated all MRI scans without access to neuropsychological results or group labels. They resolved discrepant ratings by consensus. When consensus could not be reached, the images were reviewed by a senior neurologist who was blinded to the initial ratings and relevant clinical information.

### 2.4 Eye-movement assessment

Eye movements were recorded using the EyeKnow eye-tracking system (EyeKnow; Beijing CAS-Ruiyi Information Technology Co., Ltd.). Eye-movement features were derived from overlap saccade, gap saccade, antisaccade, smooth pursuit, and lateral fixation paradigms. The extracted features characterized key domains of oculomotor function, including saccadic performance, pursuit tracking, antisaccade correction, lateral-gaze deviation, fixation stability, and square-wave jerks. The full set of characters is summarized in Table 1.

**TABLE 1:** Comparison of eye-movement parameters between the two groups.

Tasks	Features	PD group (*n* = 84)	PDD group (*n* = 213)	*P-*value
Overlap saccade task	Accuracy, median [IQR]	100.00 [100.00–100.00]	100.00 [91.67–100.00]	**0.003**
	Latency, median [IQR]	353.86 [311.67–404.13]	396.18 [341.26–456.90]	**<0.001**
	Reaction time (fastest), median [IQR]	266.06 [233.39–310.87]	299.42 [244.83–365.96]	**<0.001**
	Reaction time (average), median [IQR]	416.80 [370.95–485.87]	485.10 [409.53–582.66]	**<0.001**
	Mean saccadic velocity, median [IQR]	204.28 [170.06–249.98]	185.15 [142.69–227.24]	**0.006**
	Maximum saccadic velocity, median [IQR]	444.65 [368.65–510.47]	412.86 [325.12–498.95]	**0.038**
	Overshoot count, median [IQR]	0.00 [0.00–1.00]	0.00 [0.00–0.00]	0.288
	Undershoot count, median [IQR]	1.00 [0.00–3.00]	2.00 [1.00–4.00]	**0.026**
	Multistep saccade count, median [IQR]	0.00 [0.00–0.00]	0.00 [0.00–0.00]	0.845
	Saccadic gain, median [IQR]	0.98 [0.93–1.00]	0.95 [0.89–0.99]	**<0.001**
	Maximum amplitude (up), median [IQR]	15.80 [15.04–17.13]	15.52 [14.57–17.04]	0.095
	Maximum amplitude (down), median [IQR]	16.49 [15.53–18.03]	17.19 [15.72–20.18]	0.097
	Maximum amplitude (left), median [IQR]	17.15 [15.85–18.28]	16.90 [15.89–18.34]	0.685
	Maximum amplitude (right), median [IQR]	17.09 [16.21–18.64]	16.84 [15.95–18.94]	0.351
Smooth pursuit task	Initial time (*x*-axis), median [IQR]	663.58 [525.42–806.21]	686.06 [522.53–889.71]	0.370
	Tracking velocity (*x*-axis), median [IQR]	19.03 [16.29–23.06]	18.21 [15.11–22.00]	0.132
	Tracking acceleration (*x*-axis), median [IQR]	30.09 [20.48–43.82]	27.00 [17.20–40.08]	0.190
	Number of deviations (*x*-axis), median [IQR]	38.50 [16.50–95.25]	49.00 [25.00–99.00]	0.067
	Total deviation (*x*-axis), median [IQR]	185.80 [84.66–511.76]	256.78 [128.28–579.16]	0.054
	Mean deviation (*x*-axis), median [IQR]	5.18 [4.75–5.81]	5.30 [4.92–5.95]	0.113
	Overshoot events (*x*-axis), median [IQR]	12.00 [5.75–21.25]	12.00 [5.00–23.00]	0.968
	Undershoot (*x*-axis), median [IQR]	7.50 [2.00–24.50]	16.00 [5.00–32.00]	**0.015**
	Tracking gain (*x*-axis), median [IQR]	51.36 [25.56–97.06]	72.82 [36.68–116.20]	**0.007**
	Initial time (*y*-axis), median [IQR]	718.78 [598.96–902.76]	795.44 [633.55–1203.11]	**0.030**
	Tracking velocity (*y*-axis), median [IQR]	26.70 [22.54–30.27]	24.30 [15.92–30.79]	**0.033**
	Tracking acceleration (*y*-axis), median [IQR]	39.36 [25.91–50.74]	31.57 [14.13–51.95]	**0.043**
	Number of deviations (*y*-axis), median [IQR]	33.00 [18.00–48.00]	41.00 [25.00–65.00]	**0.004**
	Total deviation (>4°) (*y*-axis), median [IQR]	157.77 [86.19–250.08]	197.73 [125.29–325.94]	**0.005**
	Mean deviation (>4°) (*y*-axis), median [IQR]	4.90 [4.57–5.18]	4.96 [4.59–5.26]	0.365
	Overshoot count (*y*-axis), median [IQR]	8.00 [4.00–13.00]	7.00 [3.00–12.00]	0.476
	Undershoot count (*y*-axis), median [IQR]	17.50 [10.00–30.25]	23.00 [16.00–36.00]	**0.002**
	Tracking gain (*y*-axis), median [IQR]	18.87 [14.80–32.58]	26.23 [16.97–45.30]	**0.003**
	Initial time, median [IQR]	215.44 [126.90–574.03]	244.20 [151.74–727.73]	0.076
	Tracking velocity, median [IQR]	7.52 [2.92–28.09]	11.54 [3.22–24.64]	0.534
	Tracking acceleration, median [IQR]	25.13 [9.54–90.76]	31.90 [10.71–80.18]	0.533
	Number of deviations, median [IQR]	36.00 [25.75–51.50]	46.00 [30.00–62.00]	**0.010**
	Total deviation (>4°), median [IQR]	170.95 [116.71–263.56]	224.98 [144.51–314.96]	**0.010**
	Mean deviation (>4°), median [IQR]	4.73 [4.45–5.02]	4.84 [4.58–5.29]	**0.020**
	Overshoot count, median [IQR]	11.00 [5.00–16.00]	11.00 [5.00–18.00]	0.507
	Undershoot count, median [IQR]	22.00 [15.75–34.00]	26.00 [17.00–40.00]	0.073
	Tracking gain, median [IQR]	18.77 [13.70–41.38]	29.07 [15.66–61.05]	**0.006**
Anti-saccade task	Accuracy, median [IQR]	53.59 [19.55–69.42]	16.67 [0.00–36.36]	**<0.001**
	Latency, median [IQR]	362.71 [311.29–424.81]	361.65 [314.25–424.19]	0.899
	Reaction time (fastest), median [IQR]	255.44 [222.53–289.24]	288.54 [244.16–322.06]	**0.005**
	Reaction time (average), median [IQR]	445.48 [377.82–487.00]	448.14 [381.98–529.85]	0.207
	Error correction rate, median [IQR]	82.57 [59.70–100.00]	54.55 [12.50–90.00]	**<0.001**
	Error correction reaction time, median [IQR]	333.76 [272.56–409.31]	409.31 [344.35–496.54]	**<0.001**
	Mean saccadic velocity, median [IQR]	206.36 [175.87–241.56]	195.31 [134.33–245.06]	0.168
	Maximum saccadic velocity, median [IQR]	424.48 [354.76–494.76]	392.89 [320.76–499.15]	0.170
	Maximum amplitude, median [IQR]	19.27 [15.34–24.51]	15.76 [8.66–20.53]	**<0.001**
	Maximum amplitude (down), median [IQR]	20.10 [15.61–24.97]	17.01 [11.54–23.25]	**0.016**
	Maximum amplitude (left), median [IQR]	23.44 [18.77–28.88]	19.75 [14.16–24.75]	**<0.001**
	Maximum amplitude (right), median [IQR]	24.08 [19.28–30.24]	19.98 [12.76–25.78]	**<0.001**
Lateral fixation task	Number of deviations (>4°), median [IQR]	15.00 [9.00–29.75]	21.00 [11.00–43.00]	**0.006**
	Number of deviations (>2°), median [IQR]	37.00 [21.50–64.50]	46.00 [26.00–76.00]	0.200
	Total deviation (>4°), median [IQR]	141.77 [103.98–214.34]	168.62 [112.12–285.54]	**0.017**
	Total deviation duration (>4°), median [IQR]	3269.48 [2355.91–5075.90]	5117.17 [2853.50–9614.65]	**<0.001**
	Accuracy, median [IQR]	89.11 [83.10–92.16]	82.96 [68.00–90.50]	**<0.001**
	Number of square-wave jerks, median [IQR]	14.50 [6.00–29.75]	18.00 [9.00–39.00]	0.132
Gap saccade task	Accuracy, median [IQR]	100.00 [97.92–100.00]	100.00 [80.00–100.00]	**0.001**
	Latency, ms, median [IQR]	249.53 [232.18–290.55]	274.72 [242.40–312.78]	**0.005**
	Reaction time (fastest), median [IQR]	255.01 [222.42–267.15]	266.39 [232.52–312.07]	**0.014**
	Reaction time (average), median [IQR]	363.73 [312.58–403.14]	391.63 [341.63–468.35]	**0.002**
	Mean saccadic velocity, mean ± SD	201.77 ± 77.71	164.45 ± 79.29	**<0.001**
	Maximum saccadic velocity, median [IQR]	382.39 [336.39–453.84]	345.14 [251.69–429.51]	**0.003**
	Overshoot count, median [IQR]	0.00 [0.00–0.00]	0.00 [0.00–1.00]	0.194
	Undershoot count, median [IQR]	2.00 [0.00–3.25]	2.00 [1.00–4.00]	0.166
	Multistep saccade count, median [IQR]	0.00 [0.00–0.00]	0.00 [0.00–0.00]	0.991
	Saccadic gain, median [IQR]	0.96 [0.92–1.00]	0.95 [0.86–1.00]	0.170
	Maximum amplitude (up), median [IQR]	20.93 [19.34–22.27]	20.48 [19.06–22.30]	0.607
	Maximum amplitude (down), median [IQR]	21.09 [20.29–22.70]	21.01 [19.71–22.86]	0.607
	Maximum amplitude (left), median [IQR]	21.64 [20.71–22.65]	21.75 [20.36–23.19]	0.679
	Maximum amplitude (right), median [IQR]	21.52 [20.65–22.80]	21.47 [20.25–23.13]	0.714

Bold *P* values indicate statistically significant differences (*P* < 0.05).

### 2.5 Data analysis

Continuous data were described using the mean and standard deviation where suitable, whereas categorical data were presented as frequencies and proportions. Group comparisons for continuous variables were performed with Student’s *t*-test when distributional assumptions were met and with the Mann–Whitney U test otherwise. A two-sided *P* < 0.05 was considered statistically significant. Statistical analyses were carried out in R version 4.5.2.

### 2.6 Machine-learning model development

#### 2.6.1 Feature selection

Mini-Mental State Examination, MoCA, scores were collected as part of routine cognitive assessment and for characterization of the study cohort. To maintain a clear separation between cognitive-test information used in clinical ascertainment and the candidate digital biomarkers, no cognitive screening score was included in the predictor matrix used in the feature-selection and model-comparison workflow. To reduce redundancy among highly correlated features, correlation filtering was applied so that all remaining pairwise correlation coefficients were below 0.8. Feature selection used only the training cohort; the test cohort remained untouched until final performance assessment. To reduce selection bias from any single algorithm, we applied 14 complementary feature-selection procedures: univariate screening; least absolute shrinkage and selection operator using the minimum cross-validation (LASSO-min and LASSO-1se); recursive feature elimination based on random forest (RF-RFE) and support vector machine (SVM-RFE); variable selection using random forests (VSURF); variable selection using random forests with backward elimination (varSelRF); the Boruta algorithm; genetic algorithm combined with random forest (GA-RF) or support vector machine (GA-SVM); and simulated annealing combined with random forest (SA-RF) or support vector machine (SA-SVM). Overlap among the feature sets selected by these methods was visualized using an UpSet plot (Figure 2). Features selected by at least four of the 14 method were defined as stable consensus candidates. Movement disorder specialists from the participating centers reviewed the selected features and finalized the predictor set based on face validity and feasibility of implementation. The detection model was developed using the final set of selected features.

Age and sex were retained *a priori* as clinically relevant demographic covariates, regardless of their selection frequencies. This process initially identified clock-drawing score, education, antisaccade accuracy, mean overlap saccades completion duration, mean gap-saccades velocity, and lateral-gaze deviation >4° as candidate predictors. Because the clock-drawing score is itself a cognitive screening measure and may overlap with the clinical ascertainment of dementia, it was excluded to reduce potential incorporation bias and preserve the independent value of eye-movement assessment. The final predictor set therefore comprised age, sex, education, antisaccade accuracy, mean overlap saccade completion duration, mean gap saccade velocity, and lateral-gaze deviation exceeding >4°.

#### 2.6.2 Model development, comparisons, and evaluation

The dataset was stratified by diagnostic group and randomly split into a training set and a test set at a ratio of 75:25, yielding 222 and 75 patients, respectively. Thirteen Machine-learning models were subsequently developed to identify PDD among patients with PD: logistic regression, decision tree, LASSO, ridge regression, elastic net, k-nearest neighbors, LightGBM, random forest, XGBoost, SVM, multilayer perceptron, naive Bayes, and stacking. This comparative analysis was used to contextualize the performance of the final SVM model and to evaluate whether the predictive signal was stable across model classes. Within every cross-validation fold, we fitted preprocessing, SMOTE, feature selection and hyperparameter tuning using only the corresponding training partition, preventing information leakage. ROC AUC was used as the primary measure of discrimination. Classification performance was further assessed using accuracy, precision, recall, and F1 score. Calibration was examined with calibration curves, whereas overall prediction error was quantified using the Brier score. Decision-curve analysis was used to estimate clinical net benefit across candidate decision thresholds. The SVM was retained as the primary classifier for detailed evaluation, but it was not considered superior to all other models. It was selected because it showed balanced classification performance and because the radial basis function kernel can capture potentially nonlinear relationships and interactions without requiring these relationships to be specified in advance. We applied SHAP to decompose each SVM prediction into feature-level contributions relative to the model baseline.

## 3 Results

### 3.1 Cohort selection and baseline characteristics

Figure 1 summarizes the study flow. Of the 486 participants screened, 325 were identified as having idiopathic PD. After excluding 28 patients with incomplete modeling variables, 297 were included in the analytic cohort, comprising 213 patients with PDD and 84 with PD without dementia. The baseline characteristics of all participants are summarized in Table 2.

**TABLE 2:** Baseline demographic and clinical characteristics of the final analytic cohort.

Characteristics	Overall (*n* = 297)	PD group (*n* = 84)	PDD group (*n* = 213)	*P*-value
Demographic characteristics				
Participants, *n* (%)	297	84 (28.28)	213 (71.72)	
Age, median [IQR]	67.00 [60.00–75.00]	64.50 [59.00–71.50]	69.00 [61.00–76.00]	0.015
Height, median [IQR]	160.00 [155.00–165.00]	162.50 [156.75–168.00]	159.00 [152.00–165.00]	0.003
Weight, median [IQR]	60.00 [53.00–66.00]	60.00 [57.00–70.00]	60.00 [52.00–65.00]	0.013
BMI, median [IQR]	23.12 [21.11–25.71]	23.64 [22.04–26.05]	22.89 [20.89–25.10]	0.056
Neutrophils, median [IQR]	3.62 [2.80–4.39]	3.46 [2.65–4.07]	3.68 [2.82–4.47]	0.056
Lymphocytes, median [IQR]	1.56 [1.27–1.94]	1.54 [1.27–1.94]	1.57 [1.27–1.93]	0.765
Monocytes, median [IQR]	0.35 [0.29–0.43]	0.34 [0.29–0.40]	0.35 [0.27–0.44]	0.417
Platelets, median [IQR]	193.00 [158.00–234.00]	191.00 [156.00–228.50]	194.00 [161.00–238.00]	0.331
CRP, median [IQR]	1.44 [0.61–4.03]	1.25 [0.60–3.35]	1.60 [0.64–4.03]	0.198
MMSE, median [IQR]	23.00 [18.00–26.00]	26.00 [25.00–28.00]	21.00 [16.00–24.00]	<0.001
Neuropsychological and clinical scales				
MoCA, median [IQR]	19.00 [14.00–22.00]	24.00 [23.00–25.00]	16.00 [12.00–19.00]	<0.001
Clock drawing test, median [IQR]	2.00 [1.00–2.00]	3.00 [2.00–3.00]	1.00 [1.00–2.00]	<0.001
HAMA, median [IQR]	13.57 [9.00–17.00]	11.00 [7.75–15.00]	14.00 [10.00–18.00]	<0.001
HAMD, median [IQR]	10.00 [7.00–15.00]	8.50 [4.00–14.00]	11.00 [8.00–16.00]	<0.001
PSQI, median [IQR]	11.00 [8.00–13.00]	10.00 [7.00–12.00]	11.00 [9.00–14.00]	<0.001
Modified Hoehn and Yahr stage, median [IQR]	2.00 [2.00–2.50]	2.00 [2.00–2.50]	2.00 [2.00–2.50]	0.007
Total score, median [IQR]	59.00 [41.00–77.00]	50.00 [32.50–69.00]	61.00 [46.00–80.00]	0.001
Imaging and vascular risk factors				
Periventricular Fazekas grade, *n* (%)	35 (11.78)	13 (15.48)	22 (10.33)	0.174
Grade 1	149 (50.17)	47 (55.95)	102 (47.89)	
Grade 2	82 (27.61)	18 (21.43)	64 (30.05)	
Grade 3	31 (10.44)	6 (7.14)	25 (11.74)	
Deep Fazekas grade, *n* (%)	59 (19.87)	19 (22.62)	40 (18.78)	0.327
Grade 1	157 (52.86)	47 (55.95)	110 (51.64)	
Grade 2	53 (17.85)	14 (16.67)	39 (18.31)	
Grade 3	28 (9.43)	4 (4.76)	24 (11.27)	
History of hypertension, *n* (%)	127 (42.76)	36 (42.86)	91 (42.72)	0.983
History of diabetes, *n* (%)	42 (14.14)	16 (19.05)	26 (12.21)	0.128
History of coronary heart disease, *n* (%)	25 (8.42)	7 (8.33)	18 (8.45)	0.974
History of stroke, *n* (%)	46 (15.49)	10 (11.90)	36 (16.90)	0.284
Sleep disorder, *n* (%)	127 (42.76)	32 (38.10)	95 (44.60)	0.307
Education level, *n* (%)	41 (13.80)	16 (19.05)	25 (11.74)	<0.001
Junior high school	102 (34.34)	39 (46.43)	63 (29.58)	
Primary school	76 (25.59)	4 (4.76)	72 (33.80)	
Illiteracy	38 (12.79)	0 (0.00)	38 (17.84)	
Undergraduate	7 (2.36)	5 (5.95)	2 (0.94)	
Undergraduate or above	9 (3.03)	9 (10.71)	0 (0.00)	
High school	24 (8.08)	11 (13.10)	13 (6.10)	
MTA grade, *n* (%)	127 (42.76)	41 (48.81)	86 (40.38)	0.076
Grade 1	95 (31.99)	29 (34.52)	66 (30.99)	
Grade 2	60 (20.20)	10 (11.90)	50 (23.47)	
Grade 3	14 (4.71)	3 (3.57)	11 (5.16)	
Grade 4	1 (0.34)	1 (1.19)	0 (0.00)	
GCA grade, *n* (%)	49 (16.50)	19 (22.62)	30 (14.08)	0.286
Grade 0	1 (0.34)	0 (0.00)	1 (0.47)	
Grade 1	146 (49.16)	37 (44.05)	109 (51.17)	
Grade 2	97 (32.66)	28 (33.33)	69 (32.39)	
Grade 3	4 (1.35)	0 (0.00)	4 (1.88)	

Values are shown as median [IQR] or *n* (%).

**FIGURE 1:**
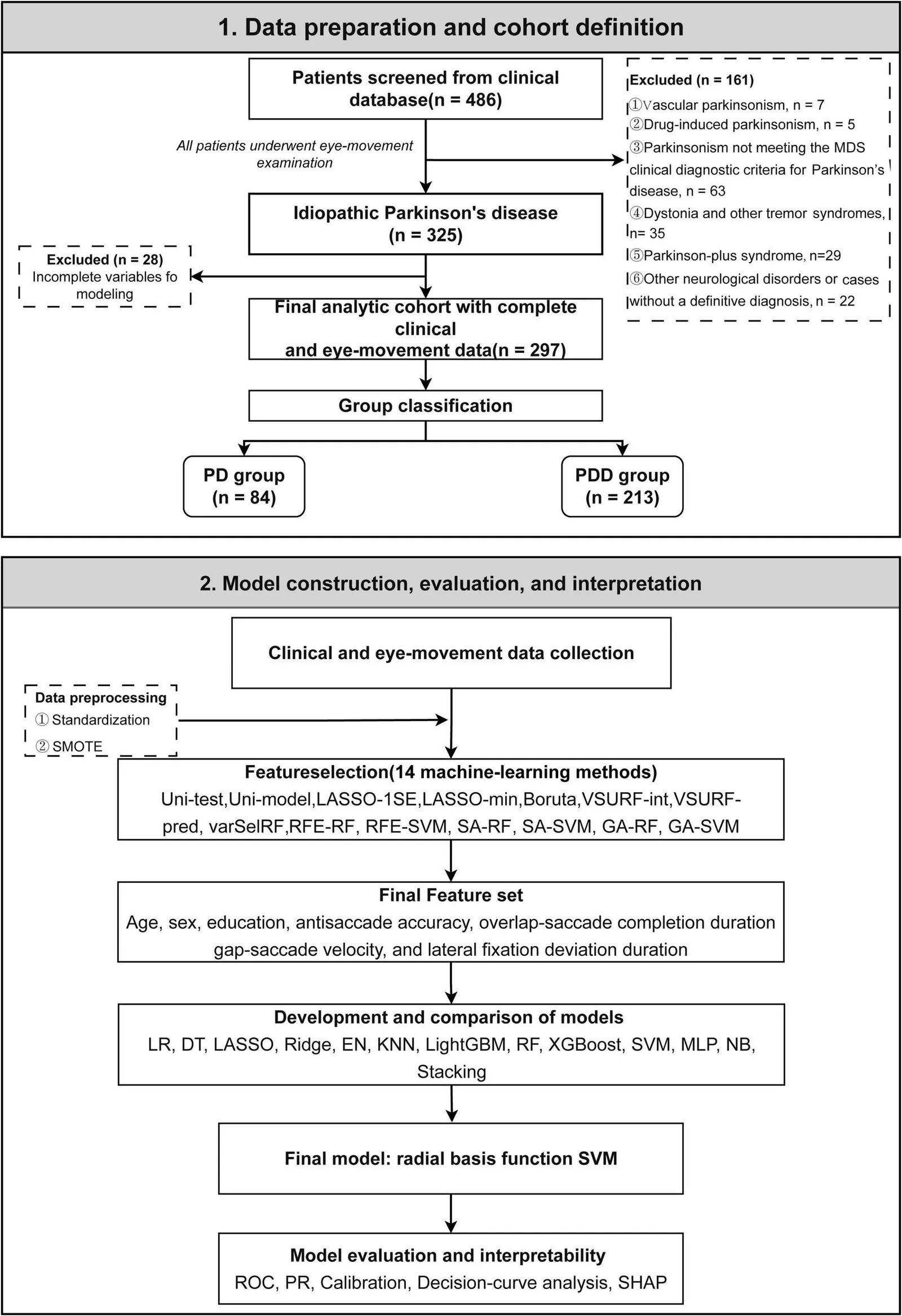
Study flowchart for participant selection, feature selection, model development, and evaluation. LR, logistic regression; DT, decision tree; LASSO, least absolute shrinkage and selection operator; Ridge, ridge regression; EN, elastic net; KNN, k-nearest neighbors; LightGBM, Light Gradient Boosting Machine; RF, random forest; XGBoost, Extreme Gradient Boosting; SVM, support vector machine; MLP, multilayer perceptron; NB, naive Bayes; Stacking, stacking ensemble.

Participants with PDD were older than those with PD without dementia (median [IQR], 69.00 [61.00–76.00] vs. 64.50 [59.00–71.50] years; *p* = 0.015). They also had lower MMSE and MoCA scores, poorer performance on the Clock Drawing Test, and higher HAMA, HAMD, and PSQI scores. They also had a higher modified Hoehn and Yahr stage, a higher MDS-UPDRS total score, and lower educational attainment (*p* < 0.05). No significant between-group differences were observed in the prevalence of hypertension, diabetes mellitus, coronary heart disease, previous stroke, or sleep disorders (all *p* > 0.05).

### 3.2 Eye-movement differences between PD and PDD groups

Eye-movement parameters differed substantially between the PD and PDD groups (Table 1 and Supplementary Table 1). In the overlap saccade task, the PDD group exhibited lower accuracy, longer latency, prolonged minimum and mean reaction times, reduced mean and peak saccadic velocities, a greater frequency of undershoots, and lower saccadic gain. During the antisaccade task, participants with PDD showed lower accuracy, a longer minimum reaction time, a lower error-correction rate, a longer error-correction reaction time, and smaller saccadic amplitudes. Significant differences were also observed in lateral fixation measures, with the PDD group demonstrating more deviations exceeding 4°, greater cumulative deviation, a longer total duration of deviations exceeding 4°, and lower fixation accuracy. In the gap saccade task, the PDD group showed lower accuracy, longer latency and reaction time, and reduced mean and peak saccadic velocities.

### 3.3 Feature selection and comparative performance of candidate models

The feature-selection procedures using 14 methods are presented in Figure 2. The candidate features identified by these methods were further reviewed by experts in Parkinson’s disease to assess their clinical relevance and feasibility of implementation. The clock-drawing score was selected by all 14 feature-selection procedures, followed by antisaccade accuracy, education, mean overlap saccade completion duration, mean gap saccade velocity, and lateral-gaze deviation >4°, which were selected by 11, 9, 6, 4, and 4 procedures, respectively. Age and sex were additionally retained as prespecified demographic covariates. After exclusion of the clock-drawing score to minimize potential overlap with cognitive outcome assessment, seven predictors were included in the final parsimonious model.

**FIGURE 2:**
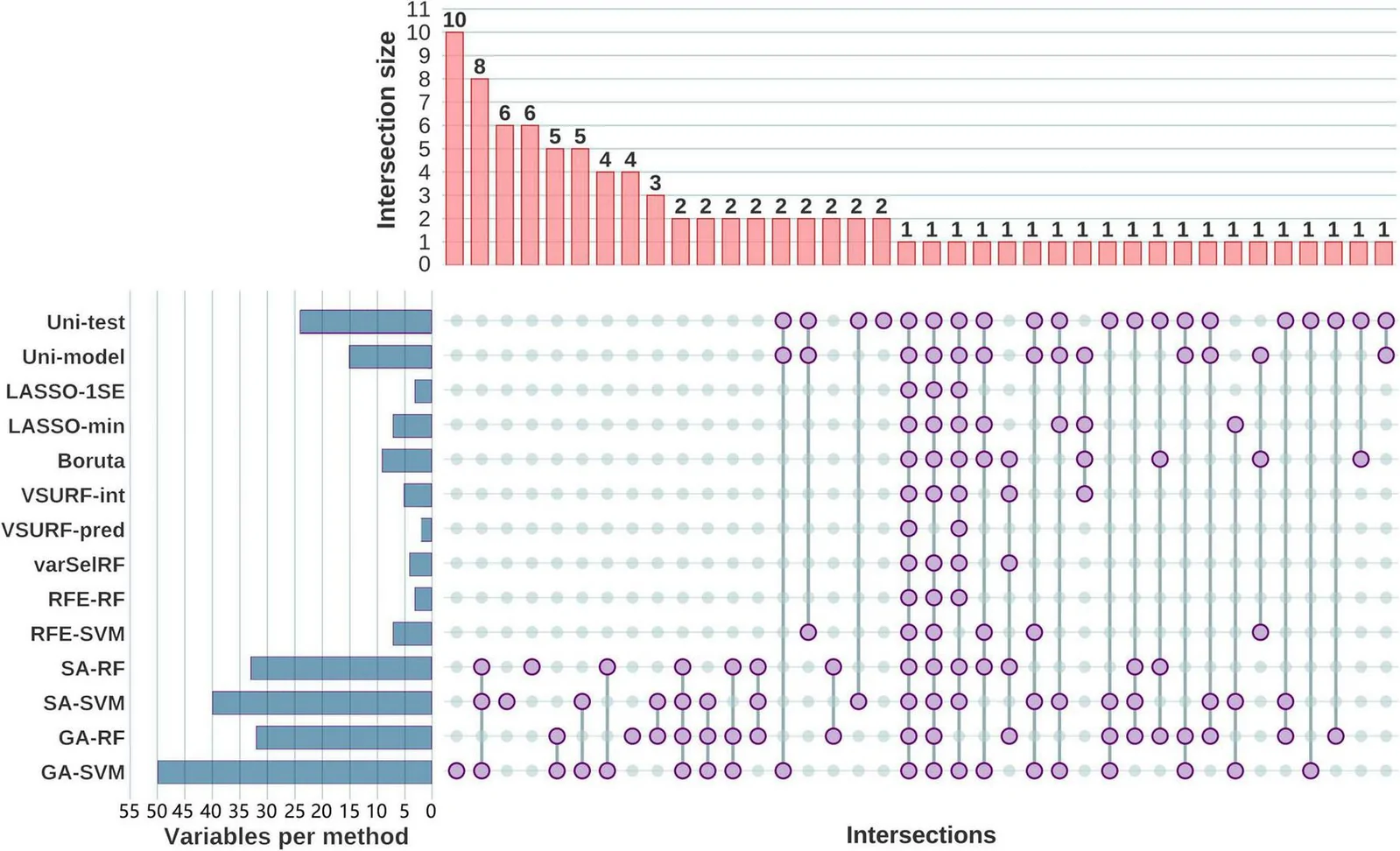
Overlap of features identified using 14 feature-selection procedures. Horizontal bars indicate the total number of variables selected by each method, whereas vertical bars indicate the size of each intersection. Filled circles connected by vertical lines denote the methods contributing to a given intersection.

Using the same seven predictors, 13 machine-learning models were developed to identify PDD among patients with PD. The candidate model set comprised logistic regression, decision tree, LASSO, ridge regression, elastic net, k-nearest neighbors, LightGBM, random forest, XGBoost, SVM, multilayer perceptron, naive Bayes, and stacking. Their performance in the held-out test set was compared across multiple discrimination and classification metrics (Figure 3 and Table 3).

**FIGURE 3:**
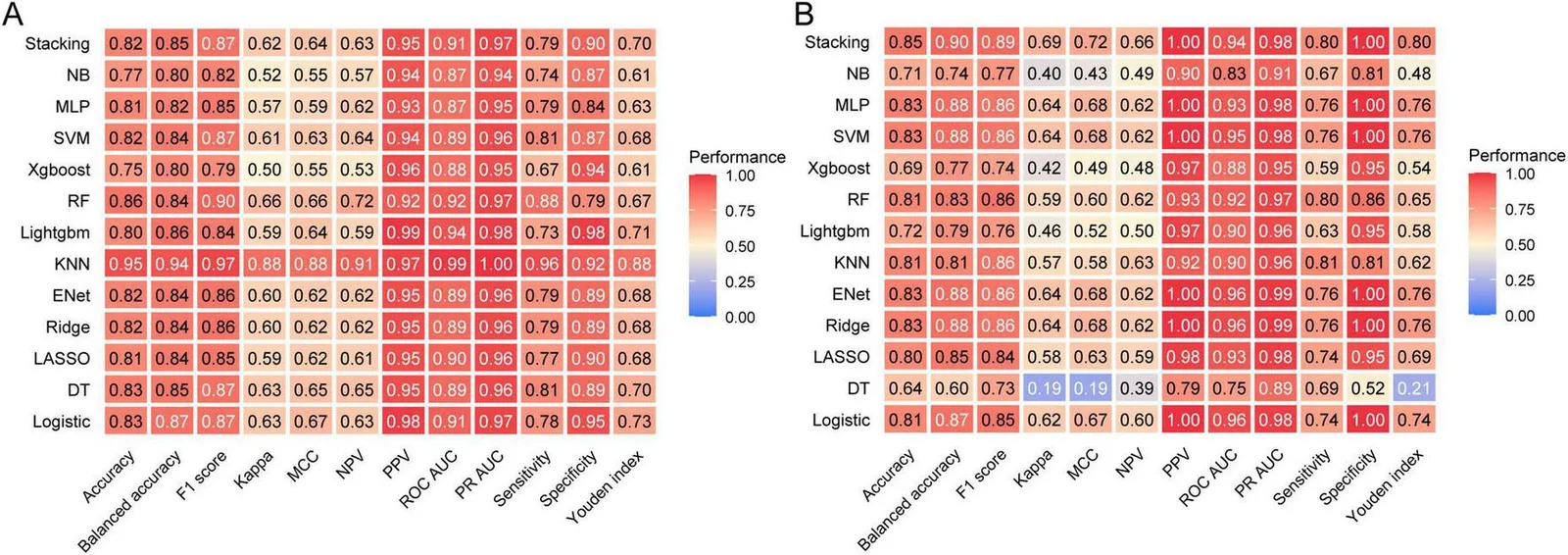
Comparative performance of machine-learning models in the training and test sets. Heatmaps show the performance of 13 candidate algorithms trained using the same selected predictors. **(A)** Prediction performance of 13 models by training cohort. **(B)** Prediction performance of 13 models by test sets.

**TABLE 3:** Seven-predictor candidate model performance in the held-out test set.

Algorithm	Test ROC AUC	Test Brier score
Logistic regression	0.962	0.077
Elastic net	0.962	0.101
Ridge regression	0.960	0.157
Support vector machine	0.947	0.102
Stacking	0.940	0.091
LASSO	0.930	0.091
Multilayer perceptron	0.930	0.174
Random forest	0.920	0.094
LightGBM	0.905	0.136
K-nearest neighbors	0.900	0.107
XGBoost	0.880	0.168
Naive Bayes	0.830	0.090
Decision tree	0.750	0.125

ROC AUC, area under the receiver operating characteristic curve.

Logistic regression and elastic net achieved the highest test-set ROC AUCs, both reaching 0.962, followed by LASSO (0.93), stacking (0.94), SVM (0.95), and multilayer perceptron (0.93). Logistic regression also yielded the lowest Brier score (0.077), followed by naive Bayes (0.090), stacking (0.091), LASSO (0.091), and random forest (0.094). Similar performance across diverse model classes suggests that the seven predictors carried reproducible signal rather than benefiting only one algorithm family. Additional ROC, precision-recall, and decision-curve analyses of the candidate models in the training and held-out test sets are presented in (Supplementary Figures 1A–F).

Logistic regression and elastic net achieved slightly higher ROC AUCs, while logistic regression had the lowest Brier score. The SVM was retained as the primary classifier for detailed evaluation because it showed a more balanced performance across sensitivity, specificity, F1 score, and Matthews correlation coefficient in the held-out test set. In addition, the radial basis function kernel can capture potentially nonlinear relationships and interactions without requiring their functional forms to be specified in advance. This choice does not imply that the SVM was consistently superior to the other models. For the final seven-feature SVM, the optimal probability threshold was 0.433, selected in the training set by maximizing the Youden index (*J* = 0.592). This threshold was then applied unchanged to the held-out test set, and the corresponding threshold-dependent performance is presented in Table 4 and Figure 4.

**TABLE 4:** Performance of the final parsimonious SVM model.

Dataset	Accuracy	Sensitivity	Specificity	PPV	NPV	F1 score	MCC	ROC AUC	PR AUC
Training set (*n* = 222)	0.824 (0.770–0.874)	0.862 (0.805–0.912)	0.730 (0.622–0.840)	0.890 (0.833–0.938)	0.676 (0.558–0.784)	0.875 (0.832–0.914)	0.579 (0.454–0.698)	0.865 (0.812–0.917)	0.936 (0.896–0.969)
Test set (*n* = 75)	0.867 (0.787–0.933)	0.870 (0.771–0.948)	0.857 (0.683–1.000)	0.940 (0.868–1.000)	0.720 (0.543–0.882)	0.904 (0.831–0.953)	0.693 (0.508–0.846)	0.947 (0.895–0.990)	0.979 (0.952–0.994)
5-fold CV	–	–	–	–	–	–	–	0.851 ± 0.048	0.935 ± 0.020

**FIGURE 4:**
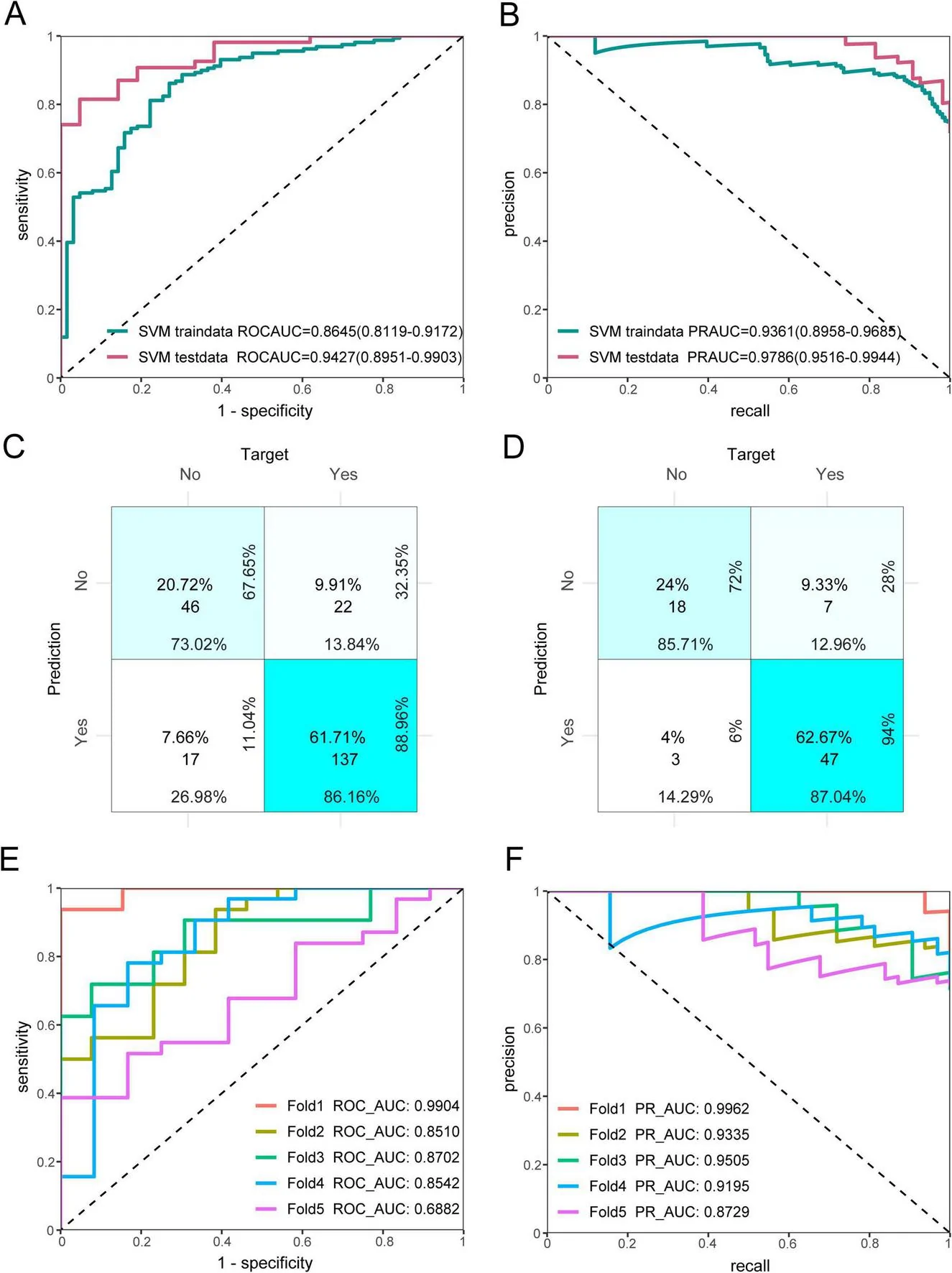
Discrimination and internal validation of the final seven-feature radial basis function SVM model. **(A)** ROC curves of diagnostic models developed by training and test cohort. **(B)** PR curves of models developed by training and test cohort. **(C)** Confusion matrix of training cohort. **(D)** Confusion matrix of test cohort. **(E)** ROC curves of diagnostic models developed by five-fold cross-validation developed by test cohort. **(F)** PR curves of diagnostic models developed by five-fold cross-validation developed by test cohort.

### 3.4 Final SVM model performance

The final seven-feature radial basis function SVM achieved balanced discrimination and threshold-dependent performance. In the training set, accuracy was 0.824, sensitivity 0.862, specificity 0.730, F1 score 0.875, ROC AUC 0.865, and PR AUC 0.936. In the held-out test set, accuracy was 0.867, sensitivity 0.870, specificity 0.857, F1 score 0.904, ROC AUC 0.947, and PR AUC 0.97 (Table 4). Five-fold cross-validation showed a mean ROC AUC of 0.851 ± 0.048 and mean PR AUC of 0.935 ± 0.020, supporting acceptable internal stability despite the modest test-set size.

ROC, precision-recall, confusion matrices, are shown in Figure 4. The high PR AUC is clinically relevant because the positive class constituted the majority of the analytic cohort. Decision curves indicated possible clinical benefit over the evaluated threshold range. Because validation was internal, this finding should be considered provisional (Supplementary Figure 1F).

### 3.5 Model interpretation

The selected predictors were clinically interpretable, and SHAP analysis was used to explain the otherwise complex decision function of the SVM model (Figure 5). Education level had the highest mean absolute SHAP value, followed by antisaccade accuracy, mean overlap-saccade completion duration, mean gap-saccade velocity, and lateral-gaze deviation duration beyond >4°. Age and sex showed comparatively low global importance. Lower antisaccade accuracy, longer mean overlap-saccade completion duration, lower mean gap-saccade velocity, and longer lateral-gaze deviation duration beyond >4° generally contributed to predictions toward PDD. The opposite feature patterns tended to contribute toward predictions of PD without dementia.

**FIGURE 5:**
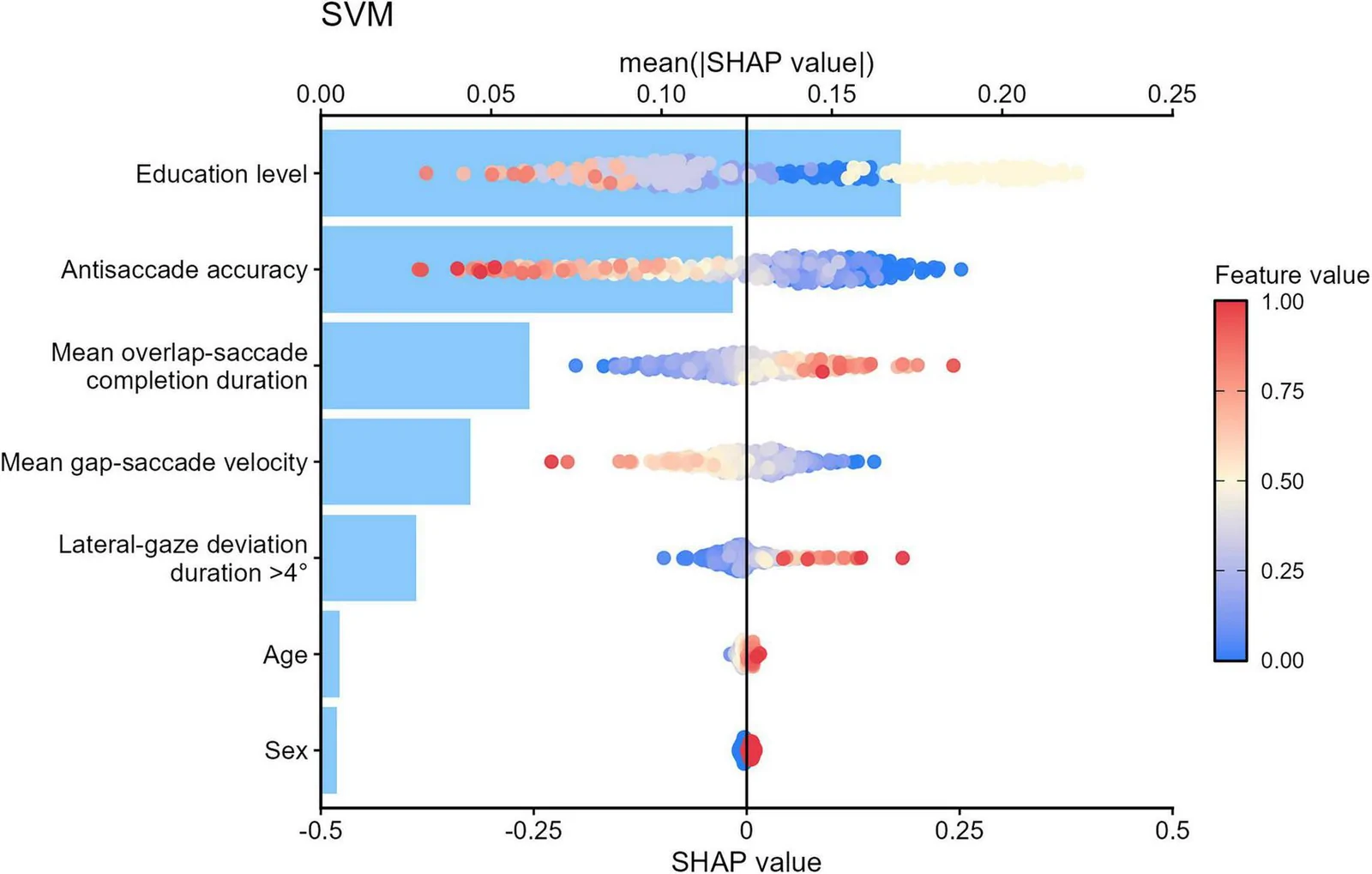
SHAP-based interpretation of the final SVM model. The plot shows global feature importance and directional feature effects for the final seven-feature SVM model. Horizontal bars indicate mean absolute SHAP values, and the accompanying beeswarm distribution shows how feature values contributed to model output across individual patients.

## 4 Discussion

This study demonstrates that combining eye-movement features with clinical information can identify dementia among patients with PD. A seven-variable model combining demographic and oculomotor measures discriminated PDD from PD in this single-center cohor. The final seven-feature SVM model achieved a test ROC AUC of 0.94 and PR AUC of 0.97, with balanced sensitivity and specificity. The most informative variables were clinically interpretable and included antisaccade accuracy, mean overlap-saccade completion duration, mean gap-saccade velocity, lateral-gaze deviation duration >4°, and education, The final SVM model was parsimonious, which may facilitate future validation, implementation, and clinical translation.

In the study, Patients with PDD were older and had fewer years of education than those without dementia. They also had lower cognitive test scores, more severe motor impairment, and higher levels of anxiety, depression, and sleep disturbance. The two groups were broadly similar in vascular risk factors and semiquantitative imaging findings. Eye-movement measures may therefore provide information beyond that obtained from routine clinical and imaging assessments.

Antisaccade accuracy exhibited one of the largest differences between the groups and was among the main contributors to model prediction. Participants must suppress a reflexive saccade toward a peripheral target and instead generate a voluntary saccade in the opposite direction. Successful performance additionally depends on retaining task instructions, shifting attention, and detecting and correcting erroneous responses (Antoniades et al., 2013). Accordingly, poorer antisaccade performance in PDD is consistent with impairment in response inhibition and executive control. PDD had longer voluntary override times during the antisaccade task than cognitively normal patients with PD and those with PD-MCI, suggesting that impaired antisaccade control may become more pronounced with increasing cognitive impairment (Brien et al., 2023). The two studies used different measures, but both point to increasing difficulty in overriding automatic eye movements as cognition worsens.

In PD, abnormalities in latency, gain, and velocity may occur in both gap and overlap saccades (Munoz et al., 2022). In the gap task, the central fixation point disappears before target onset, which facilitates the initiation of a saccade toward the peripheral target. In our study, measures derived from the gap paradigm also showed good discriminatory performance for cognitive impairment, consistent with the findings reported by Riek et al. (2023), Hao et al. (2024). By contrast, the fixation point remains visible in the overlap condition, requiring attention to be disengaged from the central stimulus before the saccade can be initiated. Overlap saccades may be more susceptible to impairment because they place greater demands on attentional disengagement and frontal–basal ganglia control. These abnormalities may be even more pronounced in PDD, where executive and attentional dysfunction is typically more severe (Munoz et al., 2022). The SHAP analysis in our study also appeared to capture this subtle distinction. Notably, rTMS treatment was associated with shorter overlap-saccade latency and completion time and higher mean saccadic velocity in patients with PD, suggesting that deficits in attentional disengagement may be at least partly reversible (Tang et al., 2024). Given that attentional and executive dysfunction commonly accompany cognitive decline in PD, measures derived from the gap and overlap tasks may be useful for cognitive screening.

Although visual fixation tasks are relatively simple, their potential clinical value should not be underestimated. Maintaining stable fixation requires sustained visual attention, stable gaze holding, and suppression of task-irrelevant saccades (Martinez-Conde et al., 2013). Li et al. (2024a) reported that patients with PD showed significantly decreased fixation accuracy in eye movement tasks, indicating potential abnormalities in visual fixation and oculomotor control. Lateral fixation showed good potential for distinguishing individuals with AD or MCI from cognitively normal controls (Qi et al., 2024). In patients with PD, excessive fixation instability or abnormal gaze shifts may reflect dysfunction of attentional control and gaze-stabilization systems. consistent with disruption of frontostriatal networks in PDD (Zgaljardic et al., 2003).

Several smooth-pursuit measures also differed between the groups, but the pattern was less consistent and none of these variables entered the final model. Smooth-pursuit performance depends on visual motion processing, continuous attention, prediction, and the method used to calculate tracking parameters. In this dataset, smooth-pursuit measures contributed less to the distinction between PD and PDD than antisaccade, gap, overlap, and fixation measures.

Previous studies have found that oculomotor abnormalities in Parkinson’s disease may be associated not only with motor dysfunction but also with cognitive decline (Brien et al., 2023; Tsitsi et al., 2023; Koch et al., 2024). Brien et al. (2023) examined 121 patients with PD using an interleaved pro-/antisaccade task and identified alterations in saccade latency, error rates, pupillary responses, and blink behavior. Their eye-tracking-based machine-learning model demonstrated favorable discriminative performance, with an AUC of 0.88, 83% sensitivity, and 78% specificity, and was associated with both MoCA and MDS-UPDRS scores (Brien et al., 2023). Similarly, Tsitsi et al. (2023) reported that, among 48 patients with PD, longer fixation duration and fewer fixations per second during reading were mainly observed in cognitively impaired patients with MoCA scores below 26.

Converging evidence has been provided by studies using other eye-movement paradigms. Koch et al. showed that tablet-based eye-tracking measures were related to cognition and disease severity, whereas Waldthaler et al. (2023) found that antisaccade errors were linked to executive dysfunction and delayed or hypometric saccades to broader cognitive impairment. Additional evidence relating blink rate and saccade duration to visuospatial memory further supports the possibility that eye-movement measures reflect cognitive as well as motor dysfunction in PD and PDD (ŞtefŞnescu et al., 2024).

The observed oculomotor abnormalities are biologically plausible. Antisaccade accuracy was substantially lower in the PDD group, consistent with impairment of inhibitory control and executive function (Waldthaler et al., 2022). Antisaccade tasks require suppression of a reflexive glance toward a peripheral stimulus and generation of a voluntary saccade in the opposite direction; this process depends on frontal executive networks, basal ganglia circuitry, and attentional control systems that are vulnerable in PD cognitive decline (Riek et al., 2023).

Although eye-movement assessment shows promise for screening cognitive decline in PD, its clinical value should not be overstated (Gibson et al., 2024; Koch et al., 2024). Eye-movement abnormalities are not specific to PDD and have also been described in Alzheimer’s disease, mild cognitive impairment, cerebrovascular disease, amyotrophic lateral sclerosis, and frontotemporal dementia (Riek et al., 2023). These measures should therefore be regarded as adjunctive screening and risk-stratification markers rather than standalone diagnostic indicators. They may complement conventional cognitive tests by objectively capturing attention, inhibitory control, and gaze stability, particularly as previous studies have reported that the MoCA may fail to identify some patients with PD-MCI (Koch et al., 2024). Therefore, Previous studies support the added value of combining eye-movement measures with cognitive, demographic, or motor variables. Koch et al. reported that tablet-based eye-movement parameters explained up to 71% of the variance in cognitive scores and distinguished mild from moderate PD with an AUC of 0.94. Li et al. (2024b) achieved validation AUCs of 0.972 by combining prosaccade velocity with gait measures and 0.861 by adding MoCA scores and education to an eye-movement model, compared with 0.726 for eye-movement features alone. Similar findings have been reported in non-PD populations, where combined gait–eye-movement models achieved AUCs of 0.987 for cognitive impairment and 0.969 for subjective cognitive decline. Collectively, these studies suggest that the main clinical value of eye tracking lies in providing complementary objective information within multivariable screening models.

Machine-learning methods can integrate diverse oculomotor measures and capture complex relationships among predictors (Cho et al., 2024). Among the 13 algorithms evaluated, logistic regression and elastic net achieved slightly higher test-set ROC AUCs than the SVM. However, the SVM showed comparable discrimination and a balanced performance across threshold-dependent metrics, and was therefore retained as the primary classifier for detailed interpretation. SHAP analysis identified education as the most influential predictor, followed by antisaccade accuracy, overlap-saccade completion duration, mean gap-saccade velocity, and lateral-gaze deviation duration >4°. The directions of these associations were consistent with the group comparisons, supporting the robustness and clinical plausibility of the selected features. By combining cross-validation with ROC, precision–recall, calibration, decision-curve, and explainability analyses, this study provides a comprehensive assessment of an interpretable model for identifying dementia within an idiopathic PD cohort.

Overall, This study extends prior eye-tracking work in PD by comparing multiple machine-learning algorithms and interpreting a parsimonious model for PDD identification in a single-center retrospective cohort. The integration of eye-tracking and clinical information requires external validation before broader clinical implementation. Future studies should further investigate the neuroanatomical relationships among the functional domains implicated in these findings.

The study also has important limitations. It was conducted at a single hospital in China, and the held-out test set was relatively small. The retrospective design may have introduced selection and information bias, and the observed associations should not be interpreted as causal. Model performance may also depend on the specific data split and may not generalize to other hospitals, eye-tracking devices, healthcare systems, or populations with different demographic and educational backgrounds.

The modest cohort size, particularly the limited number of participants with PD without dementia (*n* = 84), reduced the precision of the model performance estimates. Although SMOTE was applied to the minority PD group during model training to reduce class imbalance, it may not have fully eliminated the bias introduced by the unequal numbers of patients with PD and PDD. The predominance of PDD in the original cohort may still have affected model learning and the estimation of prevalence-dependent performance measures. Further validation in larger and more balanced external cohorts is therefore needed. We used five-fold cross-validation to examine internal performance stability. However, the limited sample size and lack of an independent cohort precluded external validation. The generalizability of the model therefore remains uncertain and should be examined in larger, multicenter populations.

Finally, eye-movement performance may be affected by visual function, calibration quality, fatigue, antiparkinsonian medication status, and understanding of task instructions. Although participants with failed calibration, poor data quality, or an inability to follow instructions were excluded, residual measurement variability cannot be excluded. Future prospective studies should standardize these factors and assess the test-retest reliability of the eye-movement measures.

This study was not designed to determine whether eye-movement features improve clinical decision-making beyond the MoCA or MMSE, either alone or in combination. The MoCA and MMSE were excluded from the predictor set because the aim was to evaluate a low-burden adjunct based on simple eye-movement tasks and readily available demographic variables. Future multicenter studies should directly compare eye-movement-only, cognitive-test-only, and combined models and assess the incremental clinical value of eye tracking.

## 5 Conclusion

Eye-movement-derived features showed internally validated discriminatory signal for identifying PDD among patients with idiopathic PD. A parsimonious seven-feature SVM model achieved favorable test-set discrimination, precision-recall performance, sensitivity, specificity, and interpretability. These findings support eye tracking as a promising digital biomarker for cognitive stratification in PD, while underscoring the need for external validation and confirmation against formal PDD diagnostic standards before clinical deployment.

## Data Availability

The original contributions presented in this study are included in this article/Supplementary material, further inquiries can be directed to the corresponding authors.
