# Supplementary material for: Eye-movement-based machine learning for identifying Parkinson’s disease dementia in a retrospective cohort: an interpretable model-development study
Source: Front Aging Neurosci. 2026 Oct 1;18:1935091. doi: 10.3389/fnagi.2026.1935091 (PMC13669113; doi:10.3389/fnagi.2026.1935091)
Supplement: Supplementary file 1 [file Data_Sheet_1.docx]

Supplementary

Table S1. Supplementary Table S1. Full comparison of eye-movement parameters between PD and PDD groups

| **Tasks** | **Features** | **PD group (n = 84)** | **PDD group (n = 213)** | **p value** |
| --- | --- | --- | --- | --- |
| Overlap saccade task | Accuracy, median [IQR] | 100.00 [100.00–100.00] | 100.00 [91.67–100.00] | 0.003 |
|  | Latency, median [IQR] | 353.86 [311.67–404.13] | 396.18 [341.26–456.90] | <0.001 |
|  | Reaction time (fastest), median [IQR] | 266.06 [233.39–310.87] | 299.42 [244.83–365.96] | <0.001 |
|  | Reaction time (average), median [IQR] | 416.80 [370.95–485.87] | 485.10 [409.53–582.66] | <0.001 |
|  | Mean saccadic velocity, median [IQR] | 204.28 [170.06–249.98] | 185.15 [142.69–227.24] | 0.006 |
|  | Maximum saccadic velocity, median [IQR] | 444.65 [368.65–510.47] | 412.86 [325.12–498.95] | 0.038 |
|  | Overshoot count, median [IQR] | 0.00 [0.00–1.00] | 0.00 [0.00–0.00] | 0.288 |
|  | Undershoot count, median [IQR] | 1.00 [0.00–3.00] | 2.00 [1.00–4.00] | 0.026 |
|  | Multistep saccade count, median [IQR] | 0.00 [0.00–0.00] | 0.00 [0.00–0.00] | 0.845 |
|  | Saccadic gain, median [IQR] | 0.98 [0.93–1.00] | 0.95 [0.89–0.99] | <0.001 |
|  | Maximum amplitude (up), median [IQR] | 15.80 [15.04–17.13] | 15.52 [14.57–17.04] | 0.095 |
|  | Maximum amplitude (down), median [IQR] | 16.49 [15.53–18.03] | 17.19 [15.72–20.18] | 0.097 |
|  | Maximum amplitude (left), median [IQR] | 17.15 [15.85–18.28] | 16.90 [15.89–18.34] | 0.685 |
|  | Maximum amplitude (right), median [IQR] | 17.09 [16.21–18.64] | 16.84 [15.95–18.94] | 0.351 |
| Smooth pursuit task | Initial time (x-axis), median [IQR] | 663.58 [525.42–806.21] | 686.06 [522.53–889.71] | 0.370 |
|  | Tracking velocity (x-axis), median [IQR] | 19.03 [16.29–23.06] | 18.21 [15.11–22.00] | 0.132 |
|  | Tracking acceleration (x-axis), median [IQR] | 30.09 [20.48–43.82] | 27.00 [17.20–40.08] | 0.190 |
|  | Number of deviations (x-axis), median [IQR] | 38.50 [16.50–95.25] | 49.00 [25.00–99.00] | 0.067 |
|  | Total deviation (x-axis), median [IQR] | 185.80 [84.66–511.76] | 256.78 [128.28–579.16] | 0.054 |
|  | Mean deviation (x-axis), median [IQR] | 5.18 [4.75–5.81] | 5.30 [4.92–5.95] | 0.113 |
|  | Overshoot count (y-axis), median [IQR] | 12.00 [5.75–21.25] | 12.00 [5.00–23.00] | 0.968 |
|  | Undershoot count (y-axis), median [IQR] | 7.50 [2.00–24.50] | 16.00 [5.00–32.00] | 0.015 |
|  | Tracking gain (x-axis), median [IQR] | 51.36 [25.56–97.06] | 72.82 [36.68–116.20] | 0.007 |
|  | Initial time (y-axis), median [IQR] | 718.78 [598.96–902.76] | 795.44 [633.55–1203.11] | 0.030 |
|  | Tracking velocity (y-axis), median [IQR] | 26.70 [22.54–30.27] | 24.30 [15.92–30.79] | 0.033 |
|  | Tracking acceleration (y-axis), median [IQR] | 39.36 [25.91–50.74] | 31.57 [14.13–51.95] | 0.043 |
|  | Number of deviations (y-axis), median [IQR] | 33.00 [18.00–48.00] | 41.00 [25.00–65.00] | 0.004 |
|  | Total deviation (>4°) (y-axis), median [IQR] | 157.77 [86.19–250.08] | 197.73 [125.29–325.94] | 0.005 |
|  | Mean deviation (>4°) (y-axis), median [IQR] | 4.90 [4.57–5.18] | 4.96 [4.59–5.26] | 0.365 |
|  | Overshoot count (y-axis), median [IQR] | 8.00 [4.00–13.00] | 7.00 [3.00–12.00] | 0.476 |
|  | Undershoot count (y-axis), median [IQR] | 17.50 [10.00–30.25] | 23.00 [16.00–36.00] | 0.002 |
|  | Tracking gain (y-axis), median [IQR] | 18.87 [14.80–32.58] | 26.23 [16.97–45.30] | 0.003 |
|  | Initial time, median [IQR] | 215.44 [126.90–574.03] | 244.20 [151.74–727.73] | 0.076 |
|  | Tracking velocity, median [IQR] | 7.52 [2.92–28.09] | 11.54 [3.22–24.64] | 0.534 |
|  | Tracking acceleration, median [IQR] | 25.13 [9.54–90.76] | 31.90 [10.71–80.18] | 0.533 |
|  | Number of deviations, median [IQR] | 36.00 [25.75–51.50] | 46.00 [30.00–62.00] | 0.010 |
|  | Total deviation (>4°), median [IQR] | 170.95 [116.71–263.56] | 224.98 [144.51–314.96] | 0.010 |
|  | Mean deviation (>4°), median [IQR] | 4.73 [4.45–5.02] | 4.84 [4.58–5.29] | 0.020 |
|  | Overshoot count, median [IQR] | 11.00 [5.00–16.00] | 11.00 [5.00–18.00] | 0.507 |
|  | Undershoot count, median [IQR] | 22.00 [15.75–34.00] | 26.00 [17.00–40.00] | 0.073 |
|  | Tracking gain, median [IQR] | 18.77 [13.70–41.38] | 29.07 [15.66–61.05] | 0.006 |
| Anti-saccade task | Accuracy, median [IQR] | 53.59 [19.55–69.42] | 16.67 [0.00–36.36] | <0.001 |
|  | Latency, median [IQR] | 362.71 [311.29–424.81] | 361.65 [314.25–424.19] | 0.899 |
|  | Reaction time (fastest), median [IQR] | 255.44 [222.53–289.24] | 288.54 [244.16–322.06] | 0.005 |
|  | Reaction time (average), median [IQR] | 445.48 [377.82–487.00] | 448.14 [381.98–529.85] | 0.207 |
|  | Error correction rate, median [IQR] | 82.57 [59.70–100.00] | 54.55 [12.50–90.00] | <0.001 |
|  | Error correction reaction time, median [IQR] | 333.76 [272.56–409.31] | 409.31 [344.35–496.54] | <0.001 |
|  | Mean saccadic velocity, median [IQR] | 206.36 [175.87–241.56] | 195.31 [134.33–245.06] | 0.168 |
|  | Maximum saccadic velocity, median [IQR] | 424.48 [354.76–494.76] | 392.89 [320.76–499.15] | 0.170 |
|  | Maximum amplitude, median [IQR] | 19.27 [15.34–24.51] | 15.76 [8.66–20.53] | <0.001 |
|  | Maximum amplitude (down), median [IQR] | 20.10 [15.61–24.97] | 17.01 [11.54–23.25] | 0.016 |
|  | Maximum amplitude (left), median [IQR] | 23.44 [18.77–28.88] | 19.75 [14.16–24.75] | <0.001 |
|  | Maximum amplitude (right), median [IQR] | 24.08 [19.28–30.24] | 19.98 [12.76–25.78] | <0.001 |
| Lateral fixation task | Number of deviations (>4°), median [IQR] | 15.00 [9.00–29.75] | 21.00 [11.00–43.00] | 0.006 |
|  | Number of deviations (>2°), median [IQR] | 37.00 [21.50–64.50] | 46.00 [26.00–76.00] | 0.200 |
|  | Total deviation (>4°), median [IQR] | 141.77 [103.98–214.34] | 168.62 [112.12–285.54] | 0.017 |
|  | Total deviation duration (>4°), median [IQR] | 3269.48 [2355.91–5075.90] | 5117.17 [2853.50–9614.65] | <0.001 |
|  | Accuracy, median [IQR] | 89.11 [83.10–92.16] | 82.96 [68.00–90.50] | <0.001 |
|  | Number of square-wave jerks, median [IQR] | 14.50 [6.00–29.75] | 18.00 [9.00–39.00] | 0.132 |
| Gap saccade task | Accuracy, median [IQR] | 100.00 [97.92–100.00] | 100.00 [80.00–100.00] | 0.001 |
|  | Latency, ms, median [IQR] | 249.53 [232.18–290.55] | 274.72 [242.40–312.78] | 0.005 |
|  | Reaction time (fastest), median [IQR] | 255.01 [222.42–267.15] | 266.39 [232.52–312.07] | 0.014 |
|  | Reaction time (average), median [IQR] | 363.73 [312.58–403.14] | 391.63 [341.63–468.35] | 0.002 |
|  | Mean saccadic velocity, mean ± SD | 201.77 ± 77.71 | 164.45 ± 79.29 | <0.001 |
|  | Maximum saccadic velocity, median [IQR] | 382.39 [336.39–453.84] | 345.14 [251.69–429.51] | 0.003 |
|  | Overshoot count, median [IQR] | 0.00 [0.00–0.00] | 0.00 [0.00–1.00] | 0.194 |
|  | Undershoot count, median [IQR] | 2.00 [0.00–3.25] | 2.00 [1.00–4.00] | 0.166 |
|  | Multistep saccade count, median [IQR] | 0.00 [0.00–0.00] | 0.00 [0.00–0.00] | 0.991 |
|  | Saccadic gain, median [IQR] | 0.96 [0.92–1.00] | 0.95 [0.86–1.00] | 0.170 |
|  | Maximum amplitude (up), median [IQR] | 20.93 [19.34–22.27] | 20.48 [19.06–22.30] | 0.607 |
|  | Maximum amplitude (down), median [IQR] | 21.09 [20.29–22.70] | 21.01 [19.71–22.86] | 0.607 |
|  | Maximum amplitude (left), median [IQR] | 21.64 [20.71–22.65] | 21.75 [20.36–23.19] | 0.679 |
|  | Maximum amplitude (right), median [IQR] | 21.52 [20.65–22.80] | 21.47 [20.25–23.13] | 0.714 |

.

Figure S1. ROC, precision-recall, and decision-curve analyses of the 13 candidate machine-learning models in the training and held-out test sets. **(A–C)** Performance in the training set, including ROC curves (A), precision-recall (PR) curves (B), and decision-curve analysis (DCA) curves (C). (**D–F)** Corresponding performance in the held-out test set, including ROC curves (D), PR curves (E), and DCA curves (F).

The candidate models included logistic regression, decision tree (DT), LASSO, ridge regression, elastic net (ENet), k-nearest neighbors (KNN), LightGBM, random forest (RF), XGBoost, support vector machine (SVM), multilayer perceptron (MLP), naive Bayes (NB), and stacking. In the DCA plots, the black and grey reference lines represent treat-all and treat-none strategies, respectively. ROC, receiver operating characteristic; PR, precision-recall; DCA, decision-curve analysis.
